# Protocol of comparison of the effects of single plasma exchange and double filtration plasmapheresis on peripheral lymphocyte phenotypes in patients with Chronic Inflammatory Demyelinating Polyradiculoneuropathy: a monocentric prospective study with single-case experimental design

**DOI:** 10.1186/s12883-022-02816-w

**Published:** 2022-08-05

**Authors:** O. Moranne, IM. Ion, R. Cezar, Z. Messikh, C. Prelipcean, S. Chkair, E. Thouvenot, Tu Anh Tran, P. Corbeau, T. Chevallier

**Affiliations:** 1 Service de Nephrologie Dialyse Apherese, Hôpital Universitaire de Nîmes, Nîmes, France; 2 Desbrest Institute of Epidemiology and Public Health UMR, INSERM - University of Montpellier, Montpellier, France; 3 Service de Neurologie, Hôpital Universitaire de Nîmes, Nîmes, France; 4 Department of Immunology, IRMB, INSERM U1183-Montpellier University, University Hospital of Nîmes, 80 Rue Augustin Fliche, 34295, Montpellier, FrancePlace du Pr Debré, 30029 Nîmes, France; 5 Department of Biostatistics, Epidemiology, IDIL (Medical Devices Evaluation), Public Health Innovation in Methodology, Nimes University Hospital, University of Montpellier, Nimes, France; 6 Institut de Génomique Fonctionnelle, Université de Montpellier, CNRS, INSERM, Montpellier, France; 7 Department of Pediatrics, Nimes University Hospital, Nimes, France INSERM U 1183, IRMB, Montpellier University, Montpellier, France; 8 Department of Immunology, Nimes University Hospital, University of Montpellier, Nimes, France

**Keywords:** CIDP, Plasma exchange, DFPP

## Abstract

**Background:**

Chronic Inflammatory Demyelinating Polyradiculoneuropathy (CIDP), a rare disorder affecting young adults, causes gradual weakness of the limbs, areflexia and impaired sensory function. New CIDP phenotypes without pathogenic antibodies but with modified cell profiles have been described. Treatments include corticotherapy, intravenous immunoglobulins, and plasmapheresis but the latter’s action mechanisms remain unclear. Plasmapheresis supposedly removes toxic agents like antibodies from plasma but it is uncertain whether it has an immune-modulating effect. Also, the refining mechanisms of the two main plasmapheresis techniques—single plasma exchange and double filtration plasmapheresis (DFPP) – are different and unclear. This study aims to compare the evolution of peripheral lymphocyte profiles in patients with CIDP according to their treatment (single centrifugation plasmapheresis or DFPP) to better grasp the action mechanisms of both techniques.

**Method:**

In this proof-of-concept, monocentric, prospective, Single-Case Experimental Design study, 5 patients are evaluated by alternating their treatment type (single plasma exchange or DFPP) for 6 courses of treatment after randomization to their first treatment type. Each course of treatment lasts 2–4 weeks. For single plasma exchange, 60 ml/kg plasma will be removed from the patient and replaced with albumin solutes, with a centrifugation method to avoid the immunological reaction caused by the membrane used with the filtration method. For DFPP, 60 ml/kg plasma will be removed from the patient with a plasma separator membrane, then processed via a fractionator membrane to remove molecules of a greater size than albumin before returning it to the patient. This technique requires no substitution solutes, only 20 g of albumin to replace what would normally be lost during a session. The primary outcome is the difference between the two plasmapheresis techniques in the variation of the TH1/TH17 ratio over the period D0H0-D0H3 and D0H0-D7. Secondary outcomes include the variation in lymphocyte subpopulations at each session and between therapeutic plasmapheresis techniques, the clinical evolution, tolerance and cost of treatments.

**Discussion:**

Understanding the action mechanisms of single plasma exchange and DFPP will help us to offer the right treatment to each patient with CIPD according to efficacy, tolerance and cost.

**Trial registration:**

ClinicalTrials.gov under the no. NCT04742374 and date of registration 10 December 2020.

## Background

The incidence of Chronic Inflammatory Demyelinating Polyradiculoneuropathy (CIDP) is 1–10 cases per 100.000 in the general population [1]. It is more common in men than women (5% of all neuropathies) and worsens with age. It is defined as a neurological disorder characterized by progressive weakness of the arms and legs, with areflexia and impaired sensory function evolving over 2 months and confirmed by electromyography [2, 3]. The physiopathology of CIDP is even lesser understood as there are various mechanisms involved such as the activation of T helper lymphocytes in peripheral blood which cross the blood–brain barrier causing damage to the nerve roots and peripheral nerves, activation of the complement cascade which destroys the myelin sheath [4] or cytotoxic T lymphocytes and also Th17 cells [5]. So far, no pathogenic autoantibodies or single triggering antigens have been identified.

Treatment of CIDP relies on immunomodulatory treatments such as plasma exchange, intravenous immunoglobulin (IVIg) or immunosuppressants (1 mg/kg of corticoids per day or a 40-mg intravenous bolus from D1-D4/month). Complete remission is only obtained in 10 -15% of patients [6]. According to a systematic review published by the Cochrane Library in 2019, evidence-based medicine to evaluate these therapies is poor, with very few randomized trials available for this particular pathology. In fact, only two randomized trials using corticotherapy, two trials for plasma exchanges and 5 trials for IVIg have been reported with a very small number of patients overall [6]. According to the recommendations of the American Society for Apheresis [7], plasma exchanges, IVIg or corticotherapy may all be used as first-line treatment depending on their availability, cost and the center’s experience or if all other therapeutic options have failed. In practice, IVIg and corticotherapy are most often used, with IVIg perhaps leading to a faster response at 6 months and corticotherapy leading to more side-effects [5, 8, 9].

Furthermore, as no pathogenic agents have been clearly identified for the majority of patients with CIDP, the action mechanisms of IVIg and therapeutic plasmapheresis are not fully understood. It has been evoked that IVIg may have an immune-modulating role and that plasmapheresis may have a role in refining an unknown toxin [10, 11]. Among these highly effective immunomodulation mechanisms, the alteration of lymphocyte T-cells or the induction of regulating lymphocyte T-cells have been evoked [5, 8–11]. One study on Guillain-Barré syndrome showed the efficacy of infusing IVIg with an increase in Treg cells and a decrease in Th1/Th17 cells in 5 patients [12]. The classical hypothesis on how therapeutic plasmapheresis works is that toxic plasma agents, such as antibodies, are removed and this has been well documented. Another hypothesis, that plasmapheresis acts on the immune system by modifying the phenotype of peripheral lymphocytes, has been suggested but less studied [13].

Besides, there are two main techniques for therapeutic plasmapheresis. The first, non-selective technique, known as “single plasma exchange”, consists of removing a certain volume of plasma, most often via a centrifugation technique without filtration, which will be replaced by a concentrated albumin solute and/or fresh, frozen plasma [14]. The second, known as semi-selective plasmapheresis or Double Filtration Plasma Pheresis (DFPP), consists of processing the plasma through a filtration membrane by means of which proteins of a larger size than that of immunoglobulins can be removed and limiting the infusion of blood by-products (Albumin and perfluorochemical [PFC] emulsion) [15]. However, this latter technique can cause the immune system to react due to the blood’s contact with the filtration membrane which is not a completely biocompatible material [16]. Therefore these two techniques do not refine the same substances, with DFPP leading to more selective refining of molecules larger in size than those of albumin, and they probably do not cause the same immune responses due, at least, to the fact of filtration or contact with a non-biocompatible membrane. Furthermore, few data about the cost of these procedures are available for comparison [17]. Moreover, a few data studying modifications to the immune system during treatment with DFPP suggest possible secondary immunomodulation. In 2000, Yoshii & Shinohara demonstrated an increase in suppressor T-cells in 11 patients with Guillain-Barré syndrome treated with DFPP. In another study conducted among patients with myasthenia, an increase in IL-10 was found after a course of DFPP suggesting that the treatment had an immune-modulating mechanism [13]. In 2000, Yoshii and Shinohara also studied CD19, CD3 and total and naïve CD4 lymphocyte sub-populations in CIPD and observed a normalization of these cells after treatment with DFPP. Likewise, they observed a normalization of the NK cells’ activity without being able to differentiate the activity of treatment with DFPP or the progress of the disease (Yoshii & Shinohara 2000; Padmanabhan et al. 2019). Paradoxically, in a study in which 18 voluntary subjects had been included, a modification in the peripheral lymphocyte profile was observed at the end of the course of DFPP with an increase in the T-helper/T-suppressor ratio and in B cells in favor of an activation of the immune system which might be explained by the reaction of blood in contact with the non-biocompatible filtration membranes and therefore specific to DFPP (Kumazawa et al. 1998). This hypothesis could not be confirmed due to the absence of a control group treated by centrifugation which did not expose blood to contact with a filtration membrane. Besides, the lymphocyte phenotype was most often evaluated at the beginning and end of the course whereas, to evaluate the immune-modulating effect of IVIgs, modifications to the immunological phenotype are observed at least one week after treatment (Oaklander & Gimigliano 2019).

Finally, plasma exchanges are proposed as a therapeutic alternative to IVIg or corticotherapy for CIDP with more information about the single plasma exchange technique [18, 19]. The literature holds very little information about the role that therapeutic plasmapheresis plays in modifying lymphocyte phenotypes. There are also no data comparing the effect of single plasma exchange with centrifugation versus DFPP on peripheral lymphocytes, tolerance and cost whereas these two techniques do not refine the same substances in plasma and have an immune activation effect related to filtration via DFPP.

In this context, the purpose of the study is to compare the evolution of the peripheral lymphocyte profile in responding patients with chronic inflammatory demyelinating polyneuropathy (CIDP) on maintenance treatment with therapeutic plasmapheresis according to whether they are being treated by the single centrifugation plasmapheresis technique (T1) or by DFPP (T2), in order to better understand the action mechanisms of therapeutic plasmapheresis with medico-economic evaluation.

## Methods

### Population

The study population consists of patients with confirmed CIDP with no known pathogenic antibodies and whose condition has remained stable under therapeutic plasmapheresis with a course every 2–4 weeks without associated medical immunosuppressant treatment for at least three months carried out at the Nephrology department of Nîmes University Hospital and validated by a specialized neurologist. All patients must have given written, informed consent and be covered by a health insurance scheme.

Any patients who have been on immunosuppressive treatment for over 3 months, or who are taking part in another Category 1 study for the purpose of research involving human subjects, will not be included. Neither will patients under legal protection, guardianship or curatorship. Also, any patients who are unfit to express their consent (or for whom it is impossible give clear information) will be excluded from the study. Women who are pregnant, in labor or breastfeeding will not be included in the study either.

### Method

**Fig. 1:**
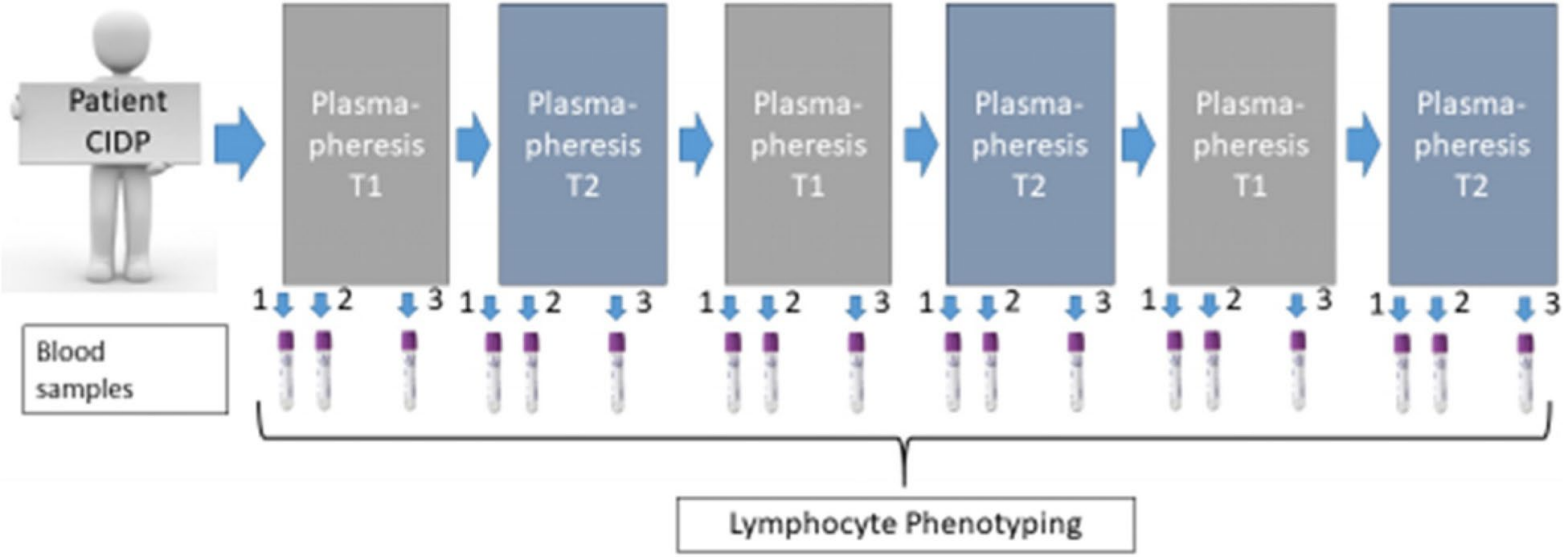
Study design

This is a proof-of-concept monocentric prospective single-case study (SCED: Single-Case Experimental Design). In our study we will proceed with the evaluation of 5 patients according to an ABABAB pattern by alternating treatment with T1 or T2 plasmapheresis for 6 cycles of treatment: T1T2/T1T2/T1T2. In order to control a possible order of attribution effect, we plan to randomize the first treatment which will either be single plasma exchange by centrifugation or DFPP. The cycles thereafter will vary accordingly (see Study Design in Fig. 1).

Each course of treatment corresponds to a period of 2 to 4 weeks. For each cycle, the lymphocyte phenotype will be evaluated before plasmapheresis (D0H0), at the end of plasmapheresis (H3) and 1 week to 10 days after treatment. Each patient will therefore generate 18 lymphocyte phenotype measures i.e. a total of 90 measures for the 5 patients participating in the study.

### Blood samples

Before the next plasmapheresis cycle (cycle T2), a blood sample will be taken in a 5 mL EDTA tube. Then the patient will undergo a plasmapheresis session and a second blood sample will be taken (5 mL EDTA tube) at the end of the session (H3). Sampling is renewed (5 mL EDTA tube) one week to ten days (D7-D10) after the session.

### Lymphocyte phenotype with flow cytometry

Lymphocyte blood cell counts (CD3 + , CD4 + , CD8 + T lymphocytes, CD19 + B lymphocytes and CD3-CD56 + NK cell) will be made in cells/μl from fresh blood samples using CYTO-STAT tetraCHROME kits with Flowcount fluorescent beads as an internal standard and tetra CXP software with a Navios cytometer (Beckman Coulter), according to the manufacturer’s instructions. Monoclonal antibodies conjugated with fluorescein isothiocyanate (FITC), phycoerythrin (PE), energy-coupled dye (ECD), PE-Cyanine5.5 (PC5.5), PE-Cyanine7 (PC7), allophycocyanin (APC), APCAlexa700, or APCAlexa750 will be purchased from Beckman Coulter and BioLegend. To measure naïve / central memory / effector CD4 + and CD8 + T-cell subsets, the antibodies will be used in the following combinations; CD197-PE/ CD45RA-ECD/ HLADR-PC7/ CD8-APC/ CD4-APCAlexa700/ CD3-APCAlexa750.

For Th1, Th2 and Th17 cells, 100 µl of total blood samples will be stained with CD4 FITC/ CXCR3-PE/ CCR6-PC5.5/ CD3-APCA750 (BioLegend). For Treg lymphocytes analysis, direct immunostaining will be performed with CD25-PE/ CD127-PC7/ FOXP3 − AF647 (clone 259D)/ CD4-APCA700/ CD3-APCA750 (BioLegend) using the PerFix-nc kit (Beckman Coulter).

Cells will be run on a Navios flow cytometer and the results analyzed with Kaluza® software (Beckman Coulter). A minimum of 20,000 lymphocytes will be gated to analyze the subpopulations. Inter-run variability will be controlled with the same batch of Rainbow 8-peak beads (Beckman Coulter).

### Therapeutic plasmapheresis techniques

#### Single plasma exchange

Single plasma exchange consists of removing plasma from the patient during the extracorporeal blood circulation session for a volume of 60 ml/kg per session and replacing the removed plasma with albumin solutes or fresh frozen plasma if there is a risk of hemorrhage (Nakanishi et al. 2014). This technique can be performed by the centrifugation method or by filtration through a filtration membrane. For the study we will do a centrifugation method which avoids the immunological reaction caused by blood coming into contact with the separation membrane. The volume of plasma processed at each course will be 60 ml/kg. For the study the material used is a COM.TEC® centrifugation machine (Frésénius Kabi, France). The intravenous fluids used will consist solely of albumin solutes except if there is a risk of hemorrhage with fresh frozen plasma added.

#### Double filtration plasmapheresis (DFPP)

Double filtration plasmapheresis consists of removing plasma from the blood with the same machine by filtering it through a separation membrane then, in a second stage, processing the extracted plasma by filtering it through a fractionator membrane whose pore size makes it possible to remove molecules of a greater size than albumin and returning the plasma to the patient without these molecules [20]. With this technique it is not necessary to infuse a substitution solute except for one flacon of albumin to provide the 20 g of albumin which would normally be lost during a session. An Infomed HF440 apparatus (Infomed SA, Suisse) will be used for the study with a “Granopen 60” (Infomed SA, Suisse) separation membrane and a “Medopen 30” (Infomed SA, Suisse) fractionator membrane. The volume of plasma processed at each session will be 60 ml/kg.

#### Clinical evaluation OF CIDP

The diagnosis of CIDP, defined as “probable” or “possible” according to the EFSN / PNS criteria, and the clinical evolution will be evaluated by a referring neurologist. This evaluation will be made before and after treatment with the following tests: the ONLS (Overall Neuropathy Limitations Scale) score, the RODS (Rasch-built Overall Disability Scale) score, the modified Rankin Scale score, the Ataxia score, the MRC score, the Hand Grip test (kg), the Nine-hole peg test (seconds) and the 100-m walking test (seconds).

#### Medico-economic study

Medico-economic studies provide data to public decision-makers to optimize balance in terms of controlling public spending, improving people's state of health and guaranteeing equitable access to healthcare [21–23]. To this aim, a health economist will estimate and compare the average cost of a session according to the two plasmapheresis techniques under evaluation. With the current system, the same French Health Insurance tariff code is used to invoice the session whichever the technique used. The study will therefore not adopt the French Health Insurance system’s viewpoint but, rather, the healthcare institution’s viewpoint as material and human resources differ and this has an impact on the cost of the sessions depending on the technique used. Thus, the criteria will be measured as follows: (i) A list of medical devices mobilized during the session will be collected. Their monetary equivalents will be found via Cpage® software (purchase price) and used for calculations. (ii) The list of blood derivatives consumed during the session will also be recorded. These data will be calculated from the nomenclature set by the French Agency for Medicines and Health Products. (iii) The length of time that nurses are mobilized for each session will be based on the number of hours required. This hourly volume will be calculated on the basis of the average annual gross salaries of this category of staff. The data needed to calculate the hourly cost of personnel will be obtained from the financial and personnel departments of the study center (from the healthcare institution's point of view).

#### Judging criteria

The primary outcome measure will be the difference between the two plasmapheresis techniques in the variation of the TH1 (CCR6-CXCR3 +) / TH17 (CCR6 + CXCR3-) ratio over the period D0H0-D0H3 and D0H0-D7/10.

Secondary outcome measures will include the evaluation of the peripheral lymphocyte sub-populations TH2 (CCR6-CXCR3-), Treg, T4, T4 HLA-DR + , T8, T8 HLA-DR + , B, naïve (CD45RA + CCR7 +), central memory cells (CD45RA-CCR7 +), and effector (CD45RA-CCR7-) T4 and T8 cells between H0 and H3 then between H0 and D7/10 globally and according to the therapeutic plasmapheresis technique used.

If no difference is observed between the two techniques, the variations in lymphocyte phenotypes will be presented for all cycles.

The evolution of each patient’s condition will be clinically evaluated between sessions with phenotyping before and after each session. There will also be specific scores for CIPD and the tolerance of each therapeutic plasmapheresis session regarding episodes of low blood pressure, dizziness or infection. The average cost of therapeutic plasmapheresis will be defined according to each technique used.

#### Sample-size calculation

For this proof-of-concept study with SCED-type methodology in conformance with the existing recommendations for this type of study [24] and, considering the rarity of the disorder under study, the inclusion of 4 to 10 patients is recommended. Therefore, 5 patients will be included.

## Discussion

This translational research study, with its original design, will help us move forwards with our understanding of the exact mechanism of how therapeutic plasmapheresis works and allow us to compare two techniques which are widely used throughout the world. Highlighting an immune-modulating effect of therapeutic plasmapheresis could provide new indications for this technique.

From a neurological viewpoint, the treatment of CIDP still represents a huge challenge involving the enrolment of only those patients with active disease and attempting to reduce or discontinue treatment to avoiding unnecessary exposure to expensive or toxic drugs. The available publications rank therapeutic plasmapheresis as the second or third line of treatment because IVIg and corticotherapy are more readily available and less invasive. However, in the light of the current health crisis provoking a huge shortage of IVIg, it has become crucial to have access to plasmapheresis, and not just as a second or third line of treatment. If there is no response to other treatments, in over 50% of cases, these patients will depend entirely on plasmapheresis. At our clinic we have adopted a side-effect profile rationale for treatment decision-making and we intend to replicate the findings from this study on a CIDP population for whom no other first line treatment is available. Moreover, proving a difference in the (hitherto undemonstrated) therapeutic efficacy of one or other of these 2 therapeutic plasmapheresis techniques, as well as tolerance and cost, would enable us improve the care of these patients.

The SCED method has been used since very early on for studies focusing on behavioral analysis, psychiatry, physiotherapy, occupational therapy or handicap [25] and has become widely developed to evaluate technological interventions [25]. This methodology resorts to individual protocols that consist of using subjects as their own controls, ensuring a quantitative approach via repeated measures. This minimizes the probability of the changes observed being the result of a non-controlled variable [24, 27, 28]. Over the last 10 years we have seen an increase in the use of SCED methodologies to evaluate health interventions based on innovating technologies [26] going as far as to produce recommendations for the publication of this type of study [29]. Thus, a user-based approach is particularly indicated for the evaluation of medical devices. Indeed an intensive, prospective study on a few individuals, using a pre-defined methodology, including systematic observations, repeated measures and appropriate data analysis is clearly indicated for this type of problem.

Single plasma exchange and DFPP may have immunomodulatory effects via various mechanisms. Plasma removal, even when replaced by frozen plasma, induces quantitative and qualitative proportions of isotypes, subclasses and glycosylation modifications in the Ig compartment that may cause immune responses at various levels [30]. Indeed, Ig effects include i) the binding of complement fragments, thereby preventing their tissue deposition [31], ii) the inhibition of lymphocyte proliferation and inflammatory cytokine production [32] iii) modulation of dendritic cell functions [33] iv) changes in inhibitory FcγRIIB expression on macrophages [34] v) an increase in the Treg/TH17 ratio [35] and vi) a modification in the diversity of lymphocytes [36].

In addition, plasma exchange also modifies the amount of soluble factors that can influence the immune system. Among these numerous factors are cytokines and chemokines. For example, the decrease in IL-12 blood concentration might reduce the Th1/Th2 ratio [37]. The same is true for DFPP concerning soluble immune mediators larger than immunoglobulins. Finally, membrane contact in the course of the filtration may also influence immune activation [18]. For all these reasons, we will monitor T-cell differentiation (naïve, memory and effector subpopulations), activation (HLA-DR expression), and CD4 + T cell subpopulations (TH1, TH2, TH17, and Treg), as well as B-cell and NK cell populations.

Finally, as far as we know, no previous studies comparing the efficacy of single plasma exchange and DFPP integrating the medico-economic aspect of these two treatments have ever been published. The results of our study should lead to a better understanding of the action mechanisms of these two techniques. Considering that these are the two main treatments available for CIDP, once we have a better grasp of them, we will be able to offer the right treatment to each patient and ultimately treat these patients in the most cost-efficient way.

## Data Availability

Not applicable.

## Abbreviations

CNIL: French data protection committee
CPP: Committee for the protection of individuals
